# Intranodal Sirolimus Induces Regulatory T Cells in Human Hepatic Lymph Nodes via Interleukin 10 Signaling

**DOI:** 10.1002/lt.26214

**Published:** 2021-07-31

**Authors:** Justin H. Nguyen, Liuyan (Jennifer) Jiang, Lu Kang, Sunita Malik, Christopher Orlando, Abba Zubair, Fatima Khwaja Rehman

**Affiliations:** 1 Division of Transplant Surgery, Department of Transplantation, Mayo Clinic, Jacksonville, FL; 2 Department of Laboratory Medicine and Pathology, Mayo Clinic, Jacksonville, FL; 3 Basic Research Unit, Mayo Clinic, Jacksonville, FL; 4 Department of Biology, University of North Florida, Jacksonville, FL

TO THE EDITOR:

Many transplant recipients die with functional transplant grafts. Immune-related morbidity and mortality attributed to conventional systemic immunosuppression continue to be the major barriers against the long-term success of solid organ transplantation including liver transplantation (LT). Targeted immunotherapy is urgently needed. To achieve the major goal of operational tolerance, one approach is to induce and expand the CD4+CD25+forkhead box P3 (FOXP3)+ regulatory T cells (Tregs).^(1,2)^ Peripheral lymph nodes are central sites for the generation of Tregs.^(3)^ Because sirolimus is known to induce and expand human Tregs in vitro as well as in vivo, we hypothesized that intranodal sirolimus induces and promotes Tregs within lymph nodes. As a first step toward nodal immunotherapy, we seek to determine if the intranodal sirolimus will induce Tregs in human hepatic lymph nodes.

We developed an 8-color flow cytometry panel to selectively measure Tregs. T cells were phenotyped with specific antibodies for CD4 (anti-CD4-fluorescein isothiocyanate [FITC], BD Pharmingen, San Diego, CA, USA, clone RPA-T4), CD3 (anti-CD3-V450, BD Horizon, BD Biosciences, San Jose, CA, USA, clone UCHT-1), CD45 (anti-CD45-V500, BD Horizon, clone HI30), CD8 (anti-CD8-allophycocyanin [APC]-H7, BD Biosciences, clone SK1), CD25 (anti-CD25-APC, BD Pharmingen, clone M-A251), and FOXP3 (anti-FOXP3-PE, BD Pharmingen, clone 259D/C7). B cells were enumerated for CD19 (anti-CD19-PE-Cy7, BD Biosciences, clone SJ25-C1). Cellular viability (7-aminoactinomycin D (7-AAD)-Percp-Cy5.5) was used for quality control, and dead cells were excluded if any cells were positive for 7-AAD. Cells ≥1 × 10^6^ per tube were prepared with BD Pharmingen stain buffer, fixed with human FOXP3 buffer, permeabilized with human FOXP3 buffer C, and conjugated with FOXP3 antibody. At least 50,000 cellular events per sample were acquired on a FacsCanto II flow cytometer (BD Biosciences). Flow Cytometry Standard format 3.0 files were exported, and data were analyzed on Kaluza version 2.1 (Beckman Coulter, Indianapolis, IN, USA). Studied cells were gated based on side scatter (SSC)/forward scatter (FSC) and CD45/side scatter (SSC) characteristics. Tregs were gated CD25+FOXP3+ events as a percentage of CD3+CD4+ cells. FOXP3 was gated at ≥10^1^ on the FOXP3 axis to ensure positive events were selected.

Hepatic lymph nodes were dissected from discarded human livers (Institutional Review Board 16–009196 and 17–007533). The hepatic lymph nodes of each liver were injected with saline or sirolimus (100 nM). They were then incubated at 37°C for 36 hours in X-vivo 15 medium (Lonza, Basel, Switzerland, catalog 04–418Q) and supplemented with 5% human serum (Sigma, St. Louis, MO, USA, catalog H4522), penicillin/streptomycin (Gibco, Waltham, MA, USA, catalog 15140–122), and 5% Glutamax (Gibco, catalog 35050–061). The 3-mm pieces of the study lymph nodes were taken and processed for processed for cytokines and chemokines using Human Cytokine Array/Chemokine Array 71-plex Discovery Assay (Millipore Sigma, Burlington, MA, USA). The remaining nodes were ground through 50-μm wire mesh, rinsed, and filtered through 50-μm nylon mesh by pipette to produce 1 mL of cell suspension, which was then processed for flow cytometry for Tregs. We found that nodal immune cells tolerated up to 36 hours of incubation well, with cellular viability of 95.1% ± 5.0% for control and 95.9% ± 6.4% for sirolimus-treated nodes. Lymphocytes, mostly T cells, were the predominant cells (Fig. 1B–F). As shown in Fig. 1A,D, we observed a significant increase in Treg proportion in sirolimus-injected hepatic lymph nodes: 1.95 ± 0.61 versus 0.61 ± 0.45 (n = 4; *P* < 0.02). On the other hand, there was no change in Tregs in the unstimulated peripheral blood monocytes by sirolimus: 0.12 ± 0.12 versus 0.01 ± 0.01 (n = 3; *P* < 0.20). These results indicated that in human hepatic lymph nodes, sirolimus alone is capable of inducing Tregs.

From the assays for cytokines and chemokines, we identified that IL18, TPO, and IL21 had 5.33 ± 4.88, 2.57 ± 0.68, and 1.45 ± 0.21 (n = 3) folds of increase in their expression levels in the sirolimus-treated lymph nodes compared with the saline controls, respectively (Fig. 2A). IL18 is essential for Treg induction; TPO and IL21 counteract Tregs. Sirolimus treatment suppressed many of the inflammatory-associated cytokines and chemokines including IL1α and IL1β. We further investigated the signaling pathways in the context of human biology using Reactome (https://reactome.org). We observed that IL10 signaling is the leading pathway in the differentiation of Tregs while suppressing inflammatory factors (Fig. 2B,C). Collectively, the results suggested that sirolimus induces cytokines that drive the formation and expansion of Tregs in human hepatic lymph nodes.

For the first time, we report here that intranodal sirolimus induces Tregs in human hepatic lymph nodes. The de novo induction and generation of Tregs in the hepatic lymph nodes are consistent with the crucial role of lymph nodes in systemic reversal of experimental multiple sclerosis,^(4)^ in Tregs generation,^(3)^ and in systemic protection against allergic conditions in humans.^(5)^ Currently, systemic infusion of Tregs that are generated ex vivo results in operational tolerance in living donor LT recipients (clinical trial UMIN-000015789).^(2)^ Monotherapy with sirolimus for 4 to 6 months induces the upregulation of Tregs and tolerogenic dendritic cells, which in turn improves operational tolerance in LT recipients (NCT02062944). These findings have paved the way for the development of ex vivo induction and expansion of tolerogenic dendritic cells in living-donor LT (NCT03164265) as well as Tregs in deceased donor LT (NCT02166177). A similar strategy of cellular immunotherapy has been performed in kidney transplantation (NCT02371434 [ONEnTreg13]). However, exogenously administered Tregs have a shortened half-life and require a large dosage of the ex vivo–generated Tregs. For the Treg immunotherapy to be clinically useful, we need a durable expansion and long-term stability of the Tregs.

Our study illustrates that the direct injection of sirolimus into a hepatic lymph node of LT recipients may provide a path toward a nodal immunotherapy eliminating or minimizing the need for systemic immunosuppressive therapy. Besides technical simplicity, de novo generation of Tregs within peripheral lymph nodes will likely ensure durability, stability, and specificity of the Tregs in their expansion.^(3)^ The immune response may occur within a few weeks or longer, such as 6 months.^(4,5)^ Repeated injections of peripheral lymph nodes may be required.^(5)^ Collectively, direct intervention into peripheral lymph nodes is a more focused and effectual approach to preemptively influence the nodal immune responses. It remains to be determined if inguinal lymph nodes are as effective as the hepatic nodes. Further investigation in a clinical trial is warranted to fully realize the role of nodal immunotherapy in LT recipients.

## Figures and Tables

**FIG. 1.**
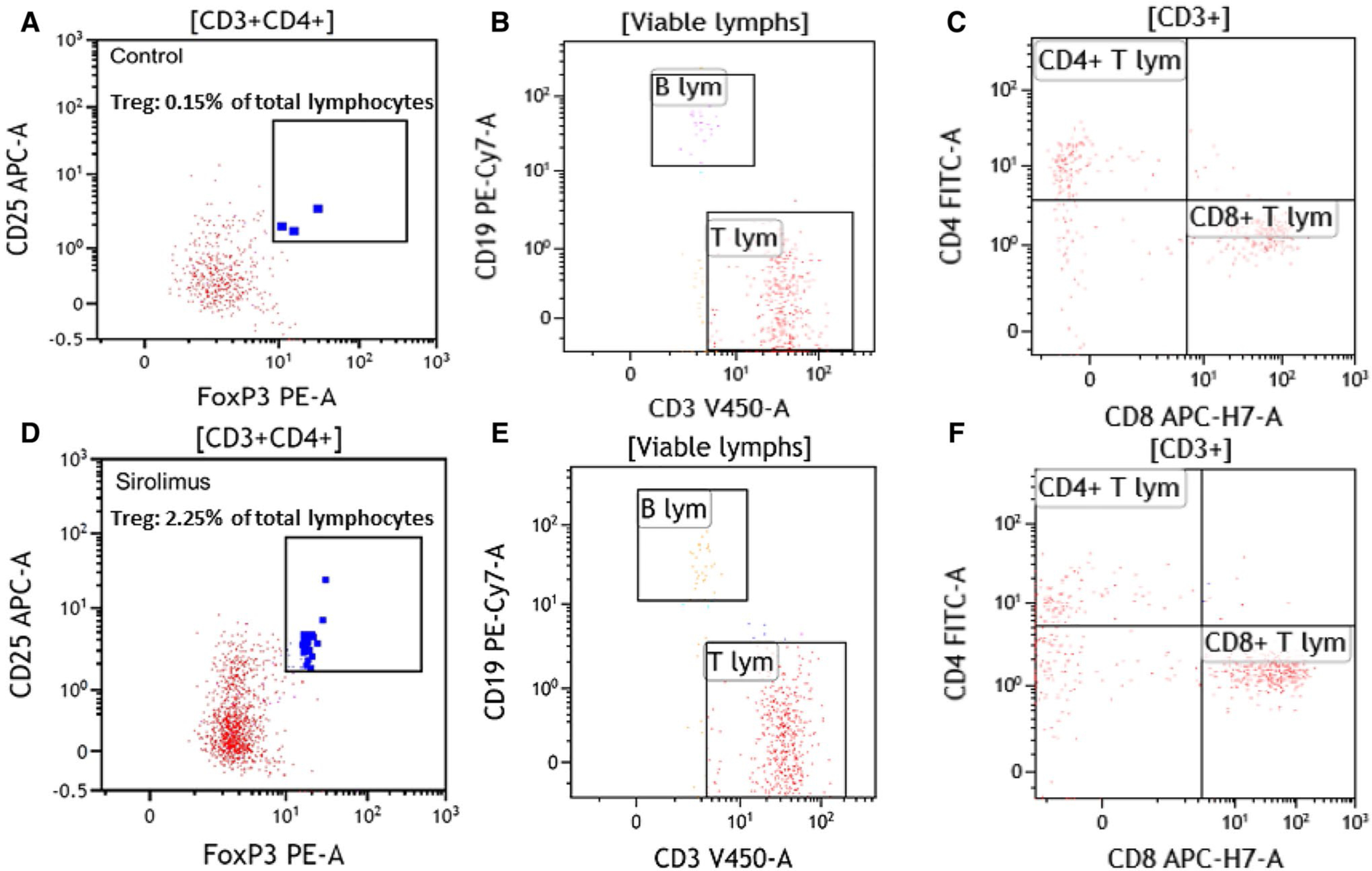
Flow cytometric assay for Tregs, B lymphocytes, T lymphocytes, and T lymphocyte subtypes. Human hepatic lymph nodes were injected (A-C) with saline as control specimen or (D-F) sirolimus alone and then incubated for 36 hours. Flow cytometry analysis identified Tregs (A and D; CD3+CD4+CD25+FOXP3+ Tregs), (B and E) CD19-positive B lymphocyte and CD3 T lymphocytes were identified, (C and F) and then CD4+ and CD8+ subtypes of T lymphocytes were further classified.

**FIG. 2.**
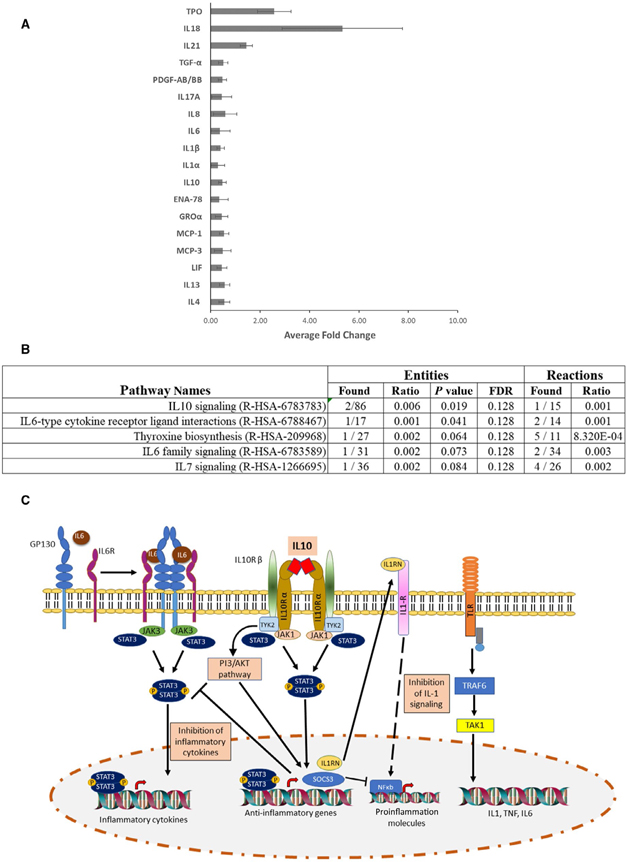
Cytokines and chemokines in human hepatic lymph nodes in response to intranodal sirolimus. (A) Differentially expressed top cytokines in the human hepatic lymph node. (B) Top signaling pathways by the cytokines in the human hepatic lymph nodes treated with intranodal sirolimus. Reactome, a curated database of pathways and reactions in human biology, was used to assess the signaling pathways. Hypergeometric distribution was used as the statistical test producing a probability score, which was corrected for FDR using the Benjamani-Hochberg method. A *P* value ≤0.05 was considered statistical significance. (C) Signaling pathways of IL10 leading to an upregulation of STAT3 along with suppressions of IL6 and IL1 signals that result in the expression of inflammatory cytokines. Abbreviations: IL18, interleukin 18; IL21, interleukin 21; IL17A, interleukin 17A; IL8, interleukin 8; IL6, interleukin 6; IL1β, interleukin 1β; IL1α, interleukin 1α; IL10, interleukin 10; MCP-1, monocyte chemoattractant protein-1; MCP-3, monocyte chemoattractant protein-3; LIF, leukemia inhibitory factor; IL13, interleukin 13; IL4, interleukin 4; IL6, interleukin 6; IL7, interleukin 7.

## Abbreviations:

7-AAD: 7-aminoactinomycin D
AKT: Protein Kinase B
APC: allophycocyanin
ENA-78: epithelial neutrophil-activating protein
FDR: false discovery rate
FITC: fluorescein isothiocyanate
FOXP3: forkhead box P3
FSC: forward scatter
GP130: glycoprotein-130
GROα: growth-regulated alpha protein
IL: interleukin
IL4: interleukin 4
IL6: interleukin 6
IL7: interleukin 7
IL8: interleukin 8
IL10: interleukin 10
IL13: interleukin 13
IL17A: interleukin 17A
IL18: interleukin 18
IL21: interleukin 21
IL1α: interleukin 1α
IL1β: interleukin 1β
JAK1: Janus Kinase 1
LIF: leukemia inhibitory factor
LT: liver transplantation
MCP-1: monocyte chemoattractant protein-1
MCP-3: monocyte chemoattractant protein-3
NF-κB: nuclear factor kappa B
P13: peptidase inhibitor 3
PDGF AB/BB: platelet-derived growth factor
SOCS3: suppressor of cytokine signaling 3
SSC: side scatter
STAT: signal transducer and activator of transcription
TAK1: transforming growth factor beta-activated kinase 1
TGF-α: transforming growth factor α
TNF: tumor necrosis factor
TPO: thyroid peroxidase
TRAF6: tumor necrosis receptor-associated factor 6
Tregs: regulatory T cells
TYK2: Tyrosine Kinase-2
